# Cardiac Dysrhythmias and Neurological Dysregulation: Manifestations of Profound Hypomagnesemia

**DOI:** 10.1155/2017/6250312

**Published:** 2017-06-05

**Authors:** Sagger Mawri, Edward Gildeh, Namita Joseph, Bobak Rabbani, Bryan Zweig

**Affiliations:** ^1^Heart & Vascular Institute, Henry Ford Hospital, Detroit, MI, USA; ^2^Department of Medicine, Henry Ford Hospital, Detroit, MI, USA

## Abstract

Magnesium is the second most common intracellular cation and serves as an important metabolic cofactor to over 300 enzymatic reactions throughout the human body. Among its various roles, magnesium modulates calcium entry and release from sarcoplasmic reticulum and regulates ATP pumps in myocytes and neurons, thereby regulating cardiac and neuronal excitability. Therefore, deficiency of this essential mineral may result in serious cardiovascular and neurologic derangements. In this case, we present the clinical course of a 76-year-old woman who presented with marked cardiac and neurological signs and symptoms which developed as a result of severe hypomagnesemia. The patient promptly responded to magnesium replacement once the diagnosis was established. We herein discuss the clinical presentation, pathophysiology, diagnosis, and management of severe hypomagnesemia and emphasize the implications of magnesium deficiency in the cardiovascular and central nervous systems. Furthermore, this case highlights the importance of having high vigilance for hypomagnesemia in the appropriate clinical setting.

## 1. Background

Magnesium plays a fundamental role in many functions of the cell including nucleic acid and protein synthesis, energy utilization, maintenance of cell membrane function, and the regulation of parathyroid hormone secretion [1, 2]. Magnesium participates in cellular second messenger systems and the regulation of vascular smooth muscle tone, thereby affecting blood pressure and altering peripheral vascular resistance [3, 4]. Magnesium is required for the stabilization of neuronal axons and plays a role in maintaining the threshold of axon stimulation [5]. Magnesium also exerts several important effects on the cardiac conduction system. It serves as an essential cofactor for the Na-K ATP pump, thereby helping to control sodium and potassium movement across cell membranes [6, 7]. Consequently, disruption in the function of this pump in the setting of hypomagnesemia impacts myocardial excitability and may result in cardiac arrhythmias [8]. Hypomagnesemia is common in the hospital setting [8, 9]. It is associated with various electrolyte derangements [10]. In severe cases, it could lead to profound clinical manifestations and serious consequences including death. Herein, we describe a unique case of an elderly woman presenting with severe neurological deficits and cardiac disturbances due to severe hypomagnesemia.

## 2. Case Presentation

A 76-year-old woman with history of hyperlipidemia, hypothyroidism, gastroesophageal reflux disease, alcohol abuse, intractable diverticular bleed 5 months earlier status after subtotal colectomy, and recently diagnosed acute right lower extremity deep venous thrombosis (off anticoagulation due to history of severe gastrointestinal bleeding) presented to the emergency department from a subacute rehabilitation center due to intermittent confusion for several days and new-onset shortness of breath of 1-hour duration.

Due to alteration in mental status, history was predominantly obtained from the patient's daughter who reported that her mother had been experiencing frequent loose bowel movements since her recent colectomy, diminished oral intake, and occasional nausea without vomiting. She reported that the patient had a recent hospitalization for dehydration and acute kidney injury at another hospital about a month ago. The patient had also been complaining of pain and tingling sensation in her feet and shaking of her arms that began about a week ago. The daughter noticed fluctuations in her mother's mental status for the past week as well. There were no recent changes in the patient's home medications which consisted of atorvastatin 10 mg daily, levothyroxine 100 mcg daily, and omeprazole 20 mg daily. The patient was not on any diuretics or laxatives prior to her hospitalization.

On presentation, the patient was afebrile, normotensive, slightly tachypneic at 22/min, tachycardic at 166/min, and saturating 96% on room air. Physical examination revealed the patient to be in moderate respiratory distress. She was noted to have dry oral mucosa and skin tenting. Lung auscultation revealed clear breath sounds bilaterally without wheezing or crackles.

Cardiovascular examination demonstrated tachycardia with an irregularly irregular rate with no murmurs and no jugular venous distention or pedal edema. Abdomen was soft, nontender, and nondistended with hyperactive bowel sounds. There was no suprapubic or flank tenderness. Neurological examination revealed the patient to be awake but alert and oriented to person only. There was evidence of resting tremors involving all extremities and impairing her speech. She had marked down-beating nystagmus and bilateral finger to nose dysmetria. There was hyperesthesia of her lower extremities and diminished vibration bilaterally distal to the tibial tuberosity. The remainder of her cranial nerve examination, strength, reflexes, and muscle tone were within normal range.

**Figure 1:**
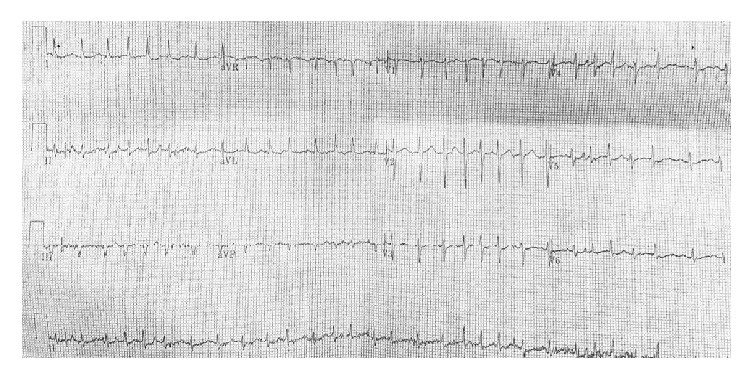
Initial ECG on presentation to the emergency department demonstrating tachycardia with an irregularly irregular rhythm and no obvious p waves consistent with atrial fibrillation with a rapid ventricular response.

An electrocardiogram (ECG) was obtained and demonstrated atrial fibrillation with rapid ventricular response at a rate of 160/min with no PR or QT interval prolongation (Figure 1). Chest X-ray revealed no acute cardiopulmonary process. Initial laboratory studies revealed an elevated white blood cell count (14,100/*μ*L), a low hemoglobin level (7.2 g/dL, near baseline), borderline potassium level (3.5 mmol/L), normal creatinine level with mildly reduced creatinine clearance (0.89 mg/dL, estimated GFR 80 mL/min/m^2^), normal glucose level, normal liver profile with low albumin level (1.9 g/dL), normal TSH level, low calcium (7.0 mg/dL) with a normal ionized calcium level (1.09 mmol/L), and unremarkable urinalysis. A 2D echocardiogram revealed preserved left ventricular function with ejection fraction of 65%, grade I diastolic dysfunction with no major valvular abnormalities, and normal atrial chamber size.

The patient was given intravenous normal saline for hydration and a bolus of intravenous diltiazem for rate control. She underwent brain computed tomography (CT) scan without contrast which revealed chronic ischemic changes without any acute intracranial hemorrhage or infarction. A lumbar puncture revealed clear central spinal fluid, normal opening pressure, no elevation in cell count or protein content, and negative gram stain and microbiology testing. Given persistent neurological deficits and concern for cerebellar injury, the patient underwent brain MRI which revealed no evidence of pathologic postcontrast enhancement to suggest cerebellar infarction and only showed chronic small vessel ischemic changes. The patient was admitted to the telemetry unit for further monitoring and work-up.

**Figure 2:**
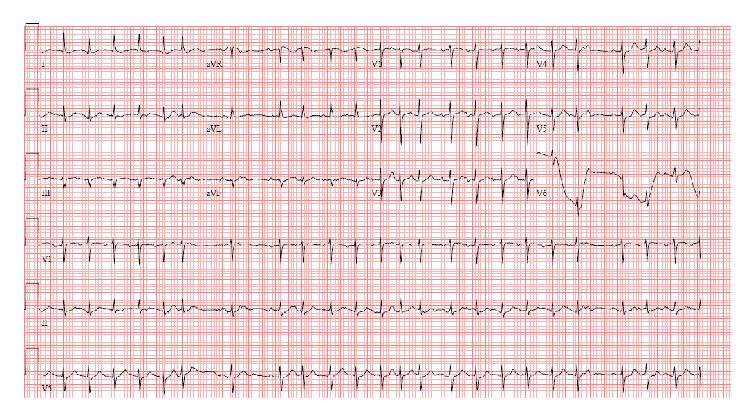
ECG in the telemetry unit demonstrating tachycardia with an irregular rhythm with presence of >3 different p morphologies consistent with multifocal atrial tachycardia (MAT).

In the telemetry ward, repeat ECG revealed the patient to be in sinus rhythm and diltiazem was discontinued. The patient's subsequent ECGs demonstrated various cardiac arrhythmias including sinus rhythm with premature atrial contractions, sinus arrhythmias, and multifocal atrial tachycardia (Figure 2) which spontaneously resolved. The patient was not noted to have frequent ventricular ectopy or nonsustained runs of ventricular tachycardia on telemetry.

Basic laboratory studies were rechecked together with an extensive panel of tests to evaluate for possible opsoclonus-myoclonus syndrome, paraneoplastic cerebellar degeneration, and vitamin deficiencies including vitamin B12, vitamin E, and thiamine (given history of alcohol abuse). Based on signs and symptoms, the patient was then started on thiamine replacement parenterally for possible Wernicke's encephalopathy. The patient was noted to have multiple loose bowel movements and thus stool analysis and clostridium difficile (C. Diff) stool assay were also checked.

Repeat laboratory investigations approximately 12 hours later demonstrated the patient to have severe magnesium deficiency (<0.5 mg/dL; normal range: 1.8–2.3 mg/dL), lower potassium level (2.9 mmol/L), and calcium level (ionized 0.98 mmol/L). It was noted that a magnesium level had not been checked previously. Aggressive magnesium replacement was initiated with intravenous magnesium sulfate 2 g every 2 hours. Within the next 12 hours, the patient's magnesium level improved to normal range (2.1 mg/dL). The patient's potassium and calcium levels normalized.

**Figure 3:**
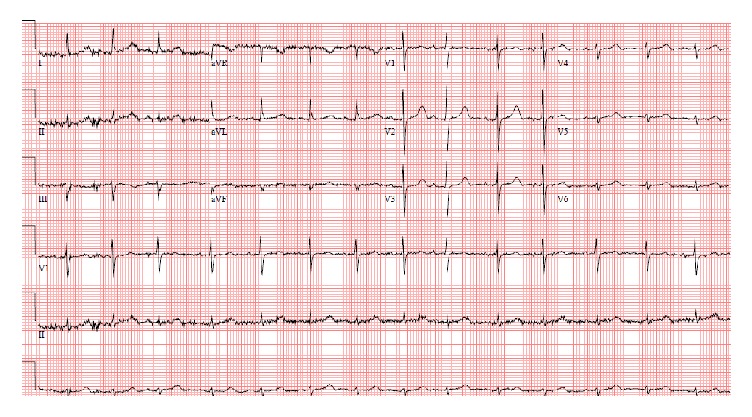
ECG after normalization of patient's magnesium level showing normal sinus rhythm.

The patient responded remarkably well to magnesium replacement with rapid clinical improvement and by the next morning, her mental status returned to baseline and she had complete resolution of her nystagmus, tremors, and remainder of the neurological signs. No further cardiac arrhythmias were noted on telemetry and all subsequent ECGs showed normal sinus rhythm (Figure 3).

Later, the patient's pending laboratory tests revealed normal thiamine level, normal vitamin B12, vitamin E, and folate levels, and completely negative paraneoplastic work-up. She had no infectious etiology for her loose stools which were determined to be a consequence of her recent colon surgery. She had no urinary evidence of magnesium wasting state. The patient's proton-pump inhibitor was discontinued and she was started on a histamine-2 receptor antagonist. She was discharged back to subacute rehabilitation center in stable condition on maintenance oral magnesium replacements.

## 3. Discussion

Magnesium is the second most common intracellular cation and is a required cofactor of over 300 enzyme systems including the Na+ - K+ - ATPase cell membrane pump found in cardiac tissue [3, 11]. Low magnesium levels decrease the activity of this pump resulting in a reduction in intracellular K+ levels, causing cell depolarization and lowering the threshold for action potential generation with prolongation of cell membrane repolarization time. This results in the increase in QTc and ultimately leads to excitability of cardiac tissue. Magnesium also acts as a physiological calcium antagonist in the myocardium with lower levels of magnesium increasing release of calcium from the L-type calcium channels of the sarcoplasmic reticulum resulting in blockade. Thus, it is a beneficial treatment in Torsades de Pointes regardless of measured magnesium levels [12]. Magnesium is also required for the stabilization of neuronal axons and plays a role in activating calcium channel in neurons. In cases of hypomagnesemia, there is reduced activation of calcium channels resulting in increased cytosolic calcium concentration which reduces the threshold of axon stimulation and causes increase in the neuromuscular excitability.

As with other ions, magnesium balance is a function of intake and excretion. There is no physiologic hormonal control of plasma magnesium and urinary magnesium excretion. Magnesium balance is achieved by absorption, mainly in the small bowel, and changes in urinary magnesium reabsorption in the loop of Henle and distal tubules of the kidney [13]. Hence, there are two major mechanisms of magnesium loss: gastrointestinal (GI) and urinary. There are continuous, unregulated GI secretory losses which contain magnesium; however, while such obligatory losses are generally mild, marked dietary deprivation may lead to progressive hypomagnesemia. GI losses most commonly occur with diarrhea rather than with vomiting, as the concentration of magnesium is higher in the lower GI tract secretions. Chronic disorders resulting in diarrhea, malabsorption, and steatorrhea as well as small bowel bypass surgery can lead to significant magnesium depletion. Recently, proton-pump inhibitors (PPIs) have come into light as a potential cause of hypomagnesemia, thought to be secondary to reduced active magnesium uptake through transient receptor potential melastatin 6 and 7 (TRPM6/7) channels present in the gut which become less active with decreased luminal pH levels [14, 15].

There are various conditions that can lead to magnesium depletion from renal causes. These include primary renal magnesium wasting disorders involving dysfunctional transport systems of magnesium with potassium and calcium, nephrotoxic agents that impair magnesium reabsorption, hypercalcemia where high calcium filtered load can diminish magnesium reabsorption, volume expansion which may hinder passive magnesium transport in the extracellular fluid, alcohol due to ethanol-induced tubular dysfunction, and diuretics [15, 16]. Both loop and thiazide diuretics can inhibit net magnesium reabsorption, while the potassium-sparing diuretics may enhance magnesium transport and reduce magnesium excretion.

Hypomagnesemia is common, occurring in about 20% of hospitalized patients and up to 65% of critically ill patients [8, 17]. Hypomagnesemia is generally asymptomatic. Symptoms commonly occur once the serum magnesium concentration falls below 1.2 mg/dL [13]. Symptoms occur due to neuromuscular irritability induced by hypomagnesemia and include muscular weakness, tremors, seizures, paresthesia, positive Chvostek and/or Trousseau signs, tetany, and a characteristic down-beating nystagmus [13, 18]. Such symptoms are congruent with those demonstrated by our patient with a magnesium level <0.5 mg/dL: opsoclonus, appendicular, and axial tremors, down-beating nystagmus, dysmetria, and encephalopathy.

Hypomagnesemia rarely occurs in isolation. It is commonly associated with other electrolyte abnormalities including hypocalcemia and hypokalemia, which confer additional ECG changes including T-wave flattening, U-waves, and widened QRS complexes. It has generally been accepted that hypomagnesemia results in increased supraventricular and ventricular dysrhythmias and incidence of Torsade's de Pointes [19]. A recent report from Tsai et al. presented a case of isolated hypomagnesemia suggesting an association with global T-wave inversions with prolonged corrected QT (QTc) interval [20].

In special patient populations, low magnesium concentrations have been associated with adverse outcomes. In acute ischemic heart disease, the incidence of ventricular arrhythmias in the first 24 hours is 2-3 times higher in patients with hypomagnesemia [21]. It has also been associated with development of atrial fibrillation in patients without underlying cardiovascular disease [22]. Such was the case with our patient who developed episodes of atrial fibrillation, sinus arrhythmia, and multifocal atrial tachycardia.

Intravenous treatment is indicated in patients with cardiac disease, convulsions, other electrolyte abnormalities, and severe hypomagnesemia (Mg2+ < 1.0 mEq/dL). Magnesium sulfate formulations should be administered with 8–12 g given over the first 24 hours and 4–6 g given per day to replace body stores [23]. Rapid repletion in hemodynamically or actively seizing patients can be accomplished with 1-2 grams (8–16 mEq) over 1 minute. Patients with a glomerular filtration rate (GFR) <30 mL/min/1.73 m^2^ are at a higher risk for hypermagnesemia and a dose reduction of 25–50% should be given with close monitoring for signs including flushing, loss of deep tendon reflexes, hypotension, AV block, and respiratory depression.

Our case demonstrates the profound clinical manifestations that may occur with severe magnesium depletion and, if left untreated, may have been fatal. Our patient's severe hypomagnesemia was thought to most likely be due to gastrointestinal losses in the setting of frequent loose stools after colectomy. The patient's nutritional status was also poor in the setting of her multiple hospitalizations and illnesses, as evident from severe hypoalbuminemia. The patient also had history of alcohol abuse before cessation five months before, further contributing to her poor nutritional status. In addition, the patient was taking daily proton-pump inhibitor which may have contributed to her magnesium loss. Each of the aforementioned contributing factors to magnesium loss was addressed accordingly and magnesium replacements were initiated with good success. We suspect the patient had been losing magnesium chronically, leading to onset of her neurological symptoms at the nursing facility and ultimately to her new-onset atrial fibrillation and other cardiac rhythm disturbances. Interestingly, there was no recorded prolongation of QT interval on ECGs, episodes of Torsade's de Pointes, or ventricular ectopy recorded during the patient's stay in the telemetry unit.

This case also illustrates the cascade of events and extensive tests that were pursued in the evaluation of our patient's marked symptomatology in the absence of an initial magnesium level in order to rule out various conditions, which happen to be in the differential diagnosis of severe hypomagnesemia. If not mistakenly forgotten during initial presentation, a serum magnesium level would have given an accurate, prompt diagnosis to explain our patient's signs and symptoms and avoid extraneous testing. Fortunately, our patient's diagnosis was established relatively quickly and the patient's clinical status normalized after correction of her hypomagnesemia. Thus, it is important to maintain a high level of vigilance for hypomagnesemia in the differential diagnosis of patients presenting with similar cardiac arrhythmias and neurological deficits, especially in those with a reason to have magnesium loss as with our patient.
